# Which are the best murine models to study Eosinophilic Chronic Rhinosinusitis? A contemporary review

**DOI:** 10.1016/j.bjorl.2023.101328

**Published:** 2023-09-15

**Authors:** Francisco Leite-Santos, Edwin Tamashiro, Adriana de Andrade Batista Murashima, Wilma T. Anselmo-Lima, Fabiana C.P. Valera

**Affiliations:** Universidade de São Paulo, Faculdade de Medicina de Ribeirão Preto, Departamento de Oftalmologia, Otorrinolaringologia e Cirurgia de Cabeça e Pescoço, Divisão de Otorrinolaringologia, Ribeirão Preto, SP, Brazil

**Keywords:** Chronic rhinosinusitis, Eosinophilic rhinosinusitis, Mouse, Nasal polyps, *Staphylococcus aureus*, *Aspergillus oryzae* protease

## Abstract

**Objective:**

Mechanisms that lead to Eosinophilic Chronic Rhinosinusitis (ECRS) are not fully established in the literature. It is desirable to assess ECRS in a model that embraces most of the related events. This article reviewed the murine models for ECRS and compared them regarding eosinophilic polypoid formation.

**Methods:**

The authors reviewed the articles that included the terms “chronic rhinosinusitis” OR “chronic sinusitis” AND “animal model”. We analyzed articles in English that evaluated both the number of polyps and the number of eosinophils in the sinus mucosa of mouse models.

**Results:**

We identified a total of 15 articles describing different models of ECRS that used BALB/c or C57BL/6 mice, and different triggers/stimulants such as *Staphylococcus aureus* Enterotoxin B (SEB) + Ovalbumin (OVA); House Dust Mite (HDM) ± Ovalbumin (OVA); and *Aspergillus oryzae* Protease (AP) + Ovalbumin (OVA). OVA associated with SEB was the commonest protocol to induce ECRS in both BALB/c and C57BL/6 mice, and it produced a robust response of eosinophilic nasal polyps in both. AP + OVA protocol also led to a good ECRS response. The other models were not considered adequate to produce eosinophilic polyps in mice.

**Conclusion:**

In conclusion, OVA associated with SEB seems to produce the most robust eosinophilic sinonasal inflammation.

## Highlights

• BALB/c mice seem to produce a more robust eosinophilic response than C57BL/6 mice.
• OVA and SEB protocol was the one that most resembled to ECRS.
• OVA and AP protocol also induced inflammation similar to ECRS, but to a lesser extent.

## Introduction

Chronic Rhinosinusitis (CRS) is characterized by inflammation of the sinonasal mucosa lasting longer than 12 weeks, leading to negative impact in the patient's quality of life.^1^ CRS poses a significant burden to both the patient and society due to its high prevalence, vast, and costly symptoms, high indirect costs, and not entirely effective treatments.^1^

The mechanisms that lead to Eosinophilic CRSwNP (ECRS) are not fully established in the literature. There is a clear need for further studies to understand ECRS physiopathology and to identify any perspective of new treatments.^2^ CRSwNP may be presented with either predominant type 1, type 2, or type 3 immune responses, and several times the inflammation may be mixed.^3^, ^4^, ^5^

Type 2 CRSwNP tend to be much more resistant to current therapies, exhibiting higher rates of recurrence than the other endotypes.^6^ It is characterized by overexpression of the cytokines IL-4, IL-5, and IL-13 and activation and recruitment of eosinophils and mast cells.^1^ Moreover, the amount of eosinophilic infiltration and the intensity of the inflammatory response are reported to be closely related to the prognosis and severity of the disease.^7^

The complexity of this pathology makes the clinical and experimental study models very problematic. Therefore, it is reasonable to assess ECRS in such a model that embraces most of the related events in a cost-effective way. The murine model has been helpful to study ECRS, because it allows the evaluation of the pattern of inflammation, epithelial remodeling, and collagen deposition.^8^ The availability of transgenic animals is useful for genetic and pathogenic studies. Finally, the murine model is the in vivo model dealing with the smallest animal, being an advantage in both ethical and financial perspectives.

However, there are limitations when transposing animal models findings to humans. The size of mice makes it challenging for studies involving surgical models or drugs involving implants and/or stents. Also, mouse nasal polyps are smaller in size and number, whereas they often occupy a considerable percentage of the nasal cavity^9^ in humans.^8^, ^9^

Over the years, several murine models for CRSwNP have been developed. In 2011, the first murine model was described in BALB/c animals, with Intranasal (IN) *Staphylococcus aureus* Enterotoxin B (SEB),^10^ and Intraperitoneal (IP) and IN Ovalbumin (OVA).^10^ Since then, several models have been proposed, in both C57BL and BALB/c animals.^9^, ^10^, ^11^, ^12^, ^13^, ^14^, ^15^, ^16^, ^17^, ^18^, ^19^, ^20^, ^21^, ^22^, ^23^, ^24^, ^25^, ^26^, ^27^, ^28^, ^29^, ^30^, ^31^, ^32^, ^33^, ^34^, ^35^ However, there is no consensus in the literature on which murine model induces a more robust eosinophilic response and a higher production of polyps.

In this review, we pool data and discuss murine models that induced ECRS presented in the literature. We also compare these studies regarding the tissue eosinophilia and the number of polypoid lesions present in the mucosa of the sinuses, which are fundamental to the evaluation of these studies.

## Methods

### Study selection

**Figure 1:**
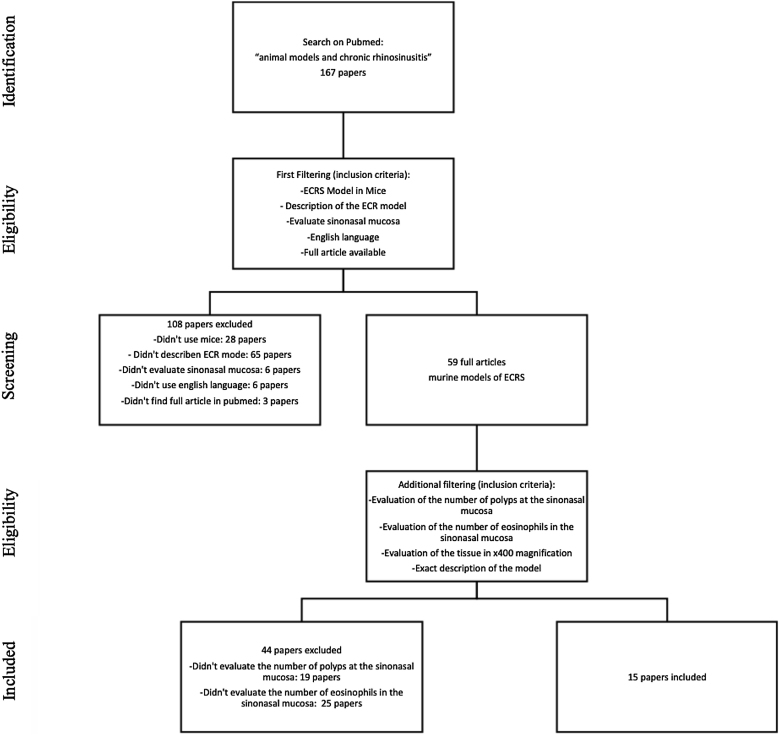
Flow of information through the different phases of the review.

We searched for English studies that described murine models of ECRS at the PubMed database. The following terms were used in the search: “animal models” and “chronic rhinosinusitis” or “chronic sinusitis”. A total of fifty-nine articles were achieved at this first research. Additional filtering was performed to this first research, accordingly: an ECRS model in mouse should be fully described; it should assess both the sinuses histology, counting both the number of polyps and the number of eosinophils on the tissue (using 400× magnification analysis); it should be written in English; should have the article available on Pubmed. Because of this assessment, a total of 44 articles were excluded: 23 described neither the number of polyps nor the number of eosinophils; 20 only evaluated the number of eosinophils, and one only evaluated the number of polyps in the sinonasal mucosa (Fig. 1).

At this stage, 15 articles were carefully read. References to relevant publications were also manually reviewed to identify additional studies. The study was conducted following the statement of preferred reporting items for systematic reviews and meta-analyses, according to PRISMA^36^ (P ‒ Murine; I ‒ Animal Model of ECRS; C ‒ Comparison; O ‒ Tissue eosinophilia and the number of polypoid lesions present in the mucosa of the sinuses).

### Data items and summary measures

The selected articles were analyzed according to mice used (BALB/c or C57BL/6) and according to the drugs used to induce eosinophilic polyps (SEB, HDM, and AP) and its combinations (SEB + HDM, SEB + OVA, AP + OVA). The dosage of each drug and the stimulus duration were also assessed. The parameters used to assess the results were the number of eosinophils and polypoid lesions in the sinonasal mucosa at histology.

Local IRB was not requested, as this is a review article.

## Results

From the selected articles, 11 induced ECRS with SEB^10^, ^14^, ^37^, ^38^, ^39^, ^40^, ^41^, ^42^, ^43^, ^44^, ^45^; 1 with AP^24^; 1 compared two models, the first induced with HDM and the latter with SEB^29^; and 2 induced ECRS with SEB in transgenic animals.^11^, ^13^ Additionally, in 5 articles, the models were used to assess different therapies to inhibit polyp formation (Resveratrol alone^37^ or associated with Mucoadhesive Nanostructured Microparticles,^14^ Cyclosporine,^40^ Chloroquine,^43^ and Tofacitinib.^44^

BALB/c mice were used in 13 experimental models (total number: 159 animals), whereas B57BL/6 were chosen in 8 (total number: 61 animals). All the animals were 4‒5-week-old at the beginning of the experimental assays.

### BALB/c animals

The same drug combination was used in all 13 experiment protocols with BALB/c mice: OVA was administered intraperitoneally, followed by intranasal OVA and SEB. Together, 159 mice were submitted to this protocol (Table 1).

**Table 1:** Articles in which eosinophilic nasal polyps were induced with an experimental model in BALB/c mice. Model A, B, and C: Ovalbumin (OVA) was administered Intraperitoneally (IP) followed by Intranasal (IN) OVA and *Staphylococcus aureus* Enterotoxin B (SEB) for 103 days. Model D: Ovalbumin (OVA) was administered Intraperitoneally (IP) followed by Intranasal (IN) OVA and *Staphylococcus aureus* Enterotoxin B (SEB) for 186 days.

Article	Number of BALB/c mice	Protocol used (drug/dosage)	Time of experiment	Number of polyps/HPF	Number of Eosinophils in the tissue
Kim et al., 2011^10^ (Model A)	15	SEB: 5 ng (IN)	103	0‒4	150
		OVA: IP: 25 µg and IN: 3%			
Kim et al., 2011^10^ (Model B)	15	SEB: 500 ng (IN)	103	0‒3	80
		OVA: IP: 25 µg and IN: 3%			
Kim et al., 2013^39^ (Model C)	20	SEB: 10 ng (IN)	103	1‒4	100‒200
		OVA: IP: 25 µg and IN: 3%			
Lee et al., 2014[47] (Model C)	8	SEB: 10 ng (IN)	103	3	50
		OVA: IP: 25 µg and IN: 3%			
Hong et al., 2015^41^ (Model C)	15	SEB: 10 ng (IN)	103	1‒2	60‒100
		OVA: IP: 25 µg and IN: 3%			
Shin et al. 2015^40^ (Model C)	10	SEB: 10 ng (IN)	103	3‒4	40‒60
		OVA: IP: 25 µg and IN: 3%			
Chang et al., 2015^42^ (Model C)	10	SEB: 10 ng	103	1‒3	40‒70
		OVA: IP: 25 µg and IN: 3%			
Kim et al., 2016^43^ (Model C)	11	SEB: 10 ng	103	2‒4	50
		OVA: IP: 25 µg and IN: 3%			
Kim et al., 2016^43^ (Model D)	8	SEB: 10 ng	186	8‒12	150
		OVA: IP: 25 µg and IN: 3%			
Lee et al., 2017^14^ (Model C)	7	SEB: 10 ng	103	4‒8	150^a^
		OVA: IP: 25 µg and IN: 3%			
Choi et al., 2020^45^ (Model C)	20	SEB: 10 ng	103	5‒20	40‒70
		OVA: IP: 25 µg and IN: 3%			
Joo et al., 2021^46^ (Model C)	10	SEB: 10 ng	103	2‒4	80‒150
		OVA: IP: 25 µg and IN: 3%			
Wee et al., 2021^38^ (Model C)	10	SEB: 10 ng	103	1‒5	20‒30
		OVA: IP: 25 µg and IN: 3%			

HPF, High Power Field.

a Mean Value.

The same dosage of IP OVA (25 µg) and the same concentration of intranasal OVA (3%) was used in all the protocols using BALB/c. Intranasal SEB dosage was defined to be 10 ng in 10 experimental groups (n = 106),^14^, ^37^, ^38^, ^40^^,^^41^, ^43^, ^44^, ^45^, ^46^ 5 ng in two experimental groups (n = 30)^10^, ^39^ and 500 ng in one study (n = 15).^10^ In 12^10^, ^14^, ^37^, ^38^, ^39^, ^40^, ^41^, ^43^, ^44^, ^45^, ^46^ of these experimental groups, the experiment lasted 103 days, while in a single experimental group,^41^ the mice were stimulated for 186 days.

In the groups that used a dose of 10 ng of SEB for 103 days,^10^, ^14^, ^37^, ^38^, ^39^, ^40^, ^41^, ^43^, ^44^, ^45^, ^46^ the number of polyps varied from 1 to 20 per field of ×400 magnification, and the eosinophil count at the tissue ranged from 20 to 200 per field. The decrease of SEB dosage to 5 ng did not considerably change the eosinophil count (which varied from 60 to 150 eosinophils per ×400 magnification field). However, it induced fewer polyps (ranging from 0 to 4).^10^ The increase of SEB dosage to 500 ng did not affect the number of polyps (0–3 per field) or the eosinophil count (mean value of 80 eosinophils per magnification field).

One single article compared two groups of BALB/c mice using the combination of OVA and SEB as previously described, but with different times of experimentation (103 vs. 186 days).^41^ The authors observed that the increase in time of experimentation led to an increase in both variables (number of polyps: 2–4 vs. 8–12; mean eosinophil count 50 vs. 150; Table 1). A more robust inflammatory response was found in the more extended protocol (186 days) when compared to the shorter one (103 days). Nevertheless, the main tissue changes were already observed after 103 days of stimulation, and that is probably why the shorter protocol was preferred by most of the authors.

In BALB/c animals, the best protocol to induce eosinophilic polyps lasted 103 days, with an intranasal SEB dosage of 10 ng. As a result, this is the most frequent protocol used to induce eosinophilic nasal polyps.^10^, ^14^, ^37^, ^38^, ^39^, ^40^, ^41^, ^43^, ^44^, ^45^, ^46^

### C57BL/6 animals and the drugs SEB and OVA

C57BL/6 mice were stimulated with IP OVA, followed by IN OVA and SEB, in three experimental protocols. Together, 15 mice were submitted to this protocol (Table 2).

**Table 2:** Articles in which eosinophilic nasal polyps were induced with an experimental model in C57BL/6 mice. Model E and F: Ovalbumin (OVA) was administered Intraperitoneally (IP) followed by Intranasal (IN) OVA and *Staphylococcus aureus* Enterotoxin B (SEB) for 103 days.

Article	Number of C57BL/6 mice	Protocol used (drug/dosage/time)	Time of experiment	Number of polyps/HPF	Number of Eosinophils in the tissue
Kim et al., 2013^11^ (Model E)	5	SEB: 10 ng	103	0‒2	30‒150
		OVA: IP: 25 µg and IN: 6%			
Khalmuratova et al. 2016^30^ (Model E)	5	SEB: 10 ng	103	0.5–1.5	60^a^
		OVA: IP: 25 µg and IN: 6%			
Bae et al., 2020^13^ (Model F)	5	SEB: 20ng	103	1‒7	400‒600
		OVA: IP: 25 µg, IN: 100 µg and 3%			

HPF, High Power Field.

a Mean Value.

All three experiments with C57BL/6 used the same dosage of OVA intraperitoneally (25 µg). Intranasal drug concentration was: 3% of OVA and 20 ng of SEB in one experimental group (n = 5)^13^; and 6% of OVA and 10 ng of SEB in two other (total n = 10).^11^, ^29^ In all three groups,^11^, ^13^, ^29^ the experiment lasted 103 days.

The increase in OVA led to an important eosinophil count at the tissue (which ranged from 30 to 150 cells per ×400 magnification field), but to a relatively low impact in polyp formation (varying from 0 to 2).^11^, ^29^ In contrast, the increase in intranasal SEB to 20 ng significantly increased the eosinophil count (from 400 to 600 eosinophils per ×400 magnification field) and the number of polyps (ranging from 1 to 7).^13^

### C57BL/6 animals and the drugs HDM and SEB

One assay administered ID and IN HDM to induce eosinophilic polyps in C57BL/6 mice (n = 5), while IN SEB was associated in the other group to the protocol (n = 5). Ten mice were included in this study, and the experiment lasted 103 days (Table 3).

**Table 3:** Articles in which eosinophilic nasal polyps were induced with an experimental model in C57BL/6 mice. Model G: House Dust Mice (HDM) was administered Intradermic (ID) followed by Intranasal (IN) for 103 days. Model H: House Dust Mice (HDM) was administered Intradermic (ID) followed by Intranasal (IN) HDM and *Staphylococcus aureus* Enterotoxin B (SEB) for 103 days.

Article	Number of C57BL6 mice	Protocol used (drug/dosage/time)	Time of experiment	Number of polyps/HPF	Number of Eosinophils in the tissue
Khalmuratova et al. 2016^30^ (Model G)	5	HDM: 100 µg ID and 20 µg IN	103	0	40
Khalmuratova et al. 2016^30^ (Model H)	5	SEB: 10 ng	103	0.5‒1.2	70
		HDM: 100 µg ID and 20 µg IN			

HPF, High Power Field.

The same dosage of HDM intradermic (100 µg) and intranasal (20 µg) OVA was used,^29^ and intranasal SEB dosage, applied in only one group, was defined to be 10 ng (n = 5).

As pointed out in Table 3, the use of only HDM induced a mean number of 40 eosinophils per ×400 magnification field, and no polyp was observed in this protocol. The association of IN SEB (10 ng) increased both the number of polyps (from 0.5 to 1.2) and the eosinophil count at the tissue (mean of 70 eosinophils per field).

Thus, the assay with only HDM could be considered a good protocol to study allergic rhinitis, but it failed to be a representative protocol to induce eosinophilic nasal polyps.

### C57BL/6 animals and the drugs AP and OVA

One study induced eosinophilic polyps in C57BL/6 mice by using the same protocol with three different periods of stimulation: 53-, 67-, and 95-days.^24^ During the assay, OVA (25 µg) was administered IP, followed by IN OVA (75 µg) and intranasal AP (2U). Together, 36 mice were submitted to this protocol (Table 4).

**Table 4:** Articles in which eosinophilic nasal polyps were induced with an experimental model in C57BL/6 mice. Model I: Ovalbumin (OVA) was administered Intraperitoneally (IP) followed by Intranasal (IN) OVA and *Aspergillus oryzae* protease (AP) for 53 days. Model J: Ovalbumin (OVA) was administered Intraperitoneally (IP) followed by intranasal (IN) OVA and *Aspergillus oryzae* protease (AP) for 67 days. Model K: Ovalbumin (OVA) was administered Intraperitoneally (IP) followed by Intranasal (IN) OVA and *Aspergillus oryzae* Protease (AP) for 95 days.

Article	Number of C57BL6 mice	Protocol used (drug/dosage/time)	Time of experiment	Number of polyps/HPF	Number of Eosinophils in the tissue
Kim et al., 2017^24^ (Model I)	12	AP: 2U	53	0	125‒150
		OVA: IP: 25 µg and IN: 75 µg			
Kim et al., 2017^24^ (Model J)	12	AP: 2U	67	0.5–1.5	125‒150
		OVA: IP: 25 µg and IN: 75 µg			
Kim et al., 2017^24^ (Model K)	12	AP: 2U	95	2–3.5	100‒125
		OVA: IP: 25 µg and IN: 75 µg			

HPF, High Power Field.

In the experimental group that lasted 53 days, no polyps were observed, and the eosinophil count at the tissue ranged from 125 to 150 per field. The increase of experimental stimulation to 67 days increased the number of polyps to 0.5–1.5 polyps per field and maintained the number of eosinophil counts (from 125 to 150 eosinophils per ×400 magnification field). In contrast, the experiment that lasted 95 days induced an increased number of polyps (from 2 to 3.5 polyps per ×400 magnification field) and maintained the number of eosinophils (from 100 to 125 eosinophils per ×400 magnification field).

## Discussion

Animal models are especially important to study the physiopathology of a specific disease (in this case, ECRS), and to evaluate the effect of possible new therapies. In this aspect, murine models of ECRS have advantages and disadvantages, like any other animal models. The major advantages of murine models are: 1) They are cheap; 2) They are easy to handle; and 3) Many reagents and antibodies are easily available. Transgenic or knockout mice, important for studies of the pathophysiological mechanisms of the disease, are also available.^11^, ^13^

In our review, the murine model most used involved BALB/c animals and the combination of IP and IN OVA with IN SEB.^10^, ^37^, ^38^, ^39^, ^40^, ^41^, ^43^, ^44^, ^45^, ^46^ The OVA concentration remained constant in all experimental groups: IP: 25 µg and IN: 3%. The ideal SEB concentration to induce eosinophilic polyps was 10 ng. The reduction of SEB concentration to 5 ng considerably decreased polyp induction,^10^ whereas the increase to 500 ng did not change the number of eosinophilic polyps per field.^10^

The duration of the models that used BALB/c animals and the combination of IP and IN OVA with IN SEB ranged from 103 to 186 days.^10^, ^37^, ^38^, ^39^, ^40^, ^41^, ^43^, ^44^, ^45^, ^46^ The duration of 186 days produced more robust eosinophilic polyps.^41^ It is important to note that the average lifespan of BALB/c mice ranges from 180 days to 365 days, and that mice would end the long-lasting protocol with around 214 days of age. With this, we conclude that the duration of the protocol of 103 days brings satisfactory results with lower chance of animal loss due to natural death.^10^, ^37^, ^38^, ^39^, ^40^, ^41^, ^43^, ^44^, ^45^, ^46^

Using the same protocol with IP and IN OVA and IN SEB, but with C57BL/6 mice, an increase of either the dose of SEB^13^ and/or OVA^11^, ^29^ was necessary to achieve a significant number of eosinophilic polyps in sinonasal mucosa. One possible reason for this is that C57BL/6 mice have attenuated allergic airway hyperresponsiveness compared to BALB/c mice, which has been demonstrated for asthma models.^8^ To achieve the same polyp and eosinophil index, the SEB dose had to be adjusted to 20 ng^13^ or the OVA concentration to 6%.^11^, ^29^ The increase of SEB seemed to be more efficient inducing ECRS in C57BL/6 mice than the increase in OVA.

Another model with C57BL/6 mice used ID and IN HDM either alone or combined with IN SEB.^29^ HDM alone did not induce polyps but stimulated eosinophilia, whereas the combination of HDM with SEB induced polyps.^29^ In summary, it seemed that the use of HDM alone can serve as a model for the study of allergic rhinitis, but not for nasal polyps. Moreover, the combination of HDM and SEB was not as efficient as OVA + SEB protocol to induce ECRS.^10^, ^37^, ^38^, ^39^, ^40^, ^41^, ^43^, ^44^, ^45^, ^46^

The combination of IN AP with IP and IN OVA was enough to produce polyps and eosinophilia,^24^ but the assays that lasted 53 and 67 days failed to produce robust nasal polyps. As the duration of the experiment increased, more polyps were observed. Possibly, if authors used the time of 103 days, they might find the same number of polyps as the combination of OVA and SEB.

In summary, IP and IN OVA associated with IN SEB is the most used protocol in both BALB/c and C57BL/6 mice to induce ECRS, as it also seems to be the most efficient to produce both polyps and eosinophils at nasal tissue. The IP and IN OVA, associated with IN AP model was also a good model to induce ECRS in C57BL/6 mice. In contrast, the combination of ID and IN HDM and IN SEB was the least efficient model to produce eosinophilic nasal polyps in mice.

## Conclusion

IP and IN OVA associated with SEB seems to produce the most robust eosinophilic sinonasal inflammation, especially in BALB/c mice.

## Funding

The present study was supported by FAPESP (process number: 2019/05843-2), and in part by the Coordenação de Aperfeiçoamento de Pessoal de Nível Superior ‒ Brasil (CAPES) ‒ Finance Code 001.

## Conflicts of interest

The authors declare no conflicts of interest.
